# Understanding the Operational Concept of a Flood-Resilient Urban Community in Jakarta, Indonesia, from the Perspectives of Disaster Risk Reduction, Climate Change Adaptation, and Development Agencies

**DOI:** 10.3390/ijerph16203993

**Published:** 2019-10-18

**Authors:** Febi Dwirahmadi, Shannon Rutherford, Dung Phung, Cordia Chu

**Affiliations:** Center for Environment and Population Health, School of Medicine, Griffith University, Brisbane 4111, Australia

**Keywords:** community resilience, flood, climate change adaptation, disaster risk reduction, development, Jakarta

## Abstract

Climate change-related extreme events such as floods have and will continue to present a great challenge to disaster risk management. There is a pressing need to develop a robust management strategy via enhancing the resiliency of the community, particularly in the context of complex urban environments, like Jakarta. Resilience is conceptualized within specific contexts and uniquely tailored to the targeted setting, yet research regarding the operational concept of a flood-resilient community in the context of Jakarta remains limited. This paper will elaborate this operational concept through understanding the desirable features and influential barriers of a flood-resilient community through the lenses of three main stakeholder groups: disaster risk reduction (DRR), climate change adaptation (CCA), and development. It will also discuss the ways in which the synergies that exist across these groups can be enhanced. Both quantitative and qualitative approaches were applied in this study, and multiple sources of data were used. The findings indicate that these groups share common views regarding the importance of human aspects being central to resilience building efforts. We argue there is an urgent need to shift the flood resilience building paradigm towards building community resilience from the people and to apply a collaborative governance approach to facilitate effective partnership between the actors involved.

## 1. Introduction

Floods have been identified as a serious global threat and have the potential to affect lives and livelihoods, change ecosystem properties, cause serious damage to agricultural and other water resource systems, spread epidemics, and have an everlasting emotional and physical effect on their human victims [1,2,3,4]. Over the last two decades, floods have accounted for 47% of all weather-related disasters, impacting over 2.1 billion people, causing major environmental disruption, and resulting in over 1 trillion USD of economic losses globally [5]. In 2018, flooding accounted for 50% of the total disaster-affected population globally [6]. Flood risks are expected to increase on the basis of climate change projections [7,8,9,10].

Given the increasing impacts of and uncertainties associated with floods, there is a pressing need to develop effective strategies to reduce the flood-related risk and vulnerability, particularly in the context of urban settings in developing countries, which are faced with uncontrolled rapid urbanization and a complex set of social and environmental threats [11,12,13]. In addition to this, the United Nations Department of Economic and Social Affairs has projected a significant increase in urban populations, particularly in lower middle-income and low-income countries, from only 41% and 32% in 2018, to 59% and 50% in 2050, respectively [14]. As more people crowd urban settings, more people will be exposed to urban flood risks [3].

In this regard, several researchers have highlighted the need to build community resilience to manage the impacts of urban flooding, e.g., [15,16,17]. Building community resilience is a bridging concept across multiple sectors, requiring a collective response from relevant key stakeholders [18]. To achieve a comprehensive and robust strategy for building community resilience for climate-related disasters (in this case—flooding), researchers have recommended a call for collaborative work between disaster risk reduction (DRR) and climate change adaptation (CCA) groups [19,20].

DRR and CCA represent two different fields that often have complimentary sets of expertise and policy goals. The former focuses more on a multi-hazard approach, learning from previous disaster events to tackle future disasters, and increasing the coping capacity, while the latter concentrates more on climate-related hazards, the projection of emerging issues associated with climate change, and increasing the adaptive capacity [21]. The implementation of DRR and CCA is not to be separated from development strategies [22], so considering the perspectives of the development sector is also relevant and important.

Although resilience building has been identified as one of the central concepts in all post-2015 global policy documents, including the Sendai Framework for Disaster Risk Reduction 2015–2030, the Paris Agreement 2015, and the Sustainable Development Goals (SDGs), which, in the global context, represent the platform for DRR, CCA, and development, respectively [23], the operational concept of urban community resilience towards flood risks is still highly contested among these stakeholders [24,25,26].

While several studies have focused on elaboration of the operational concept of community resilience in different environmental settings or different hazards like earthquake-prone areas, e.g., [27], landslide-prone areas, e.g., [28], homeland security, e.g., [29], urban infrastructure to fire hazards, e.g., [30], and urban and climate change risks, e.g., [31], coastal communities, e.g., [32], among others, floods in a complex urban setting still need to be studied. Therefore, this study is focused on understanding the operational concepts of flood resilience in an urban community within a complex setting. As a case site, the metropolitan city of Jakarta, Indonesia, which is often termed complex [33], has been selected for this study. This study is important to improving the evidence base for guiding CCA, DRR, and development practitioners in fine-tuning collaborative actions between stakeholders to build community resilience against flood risks in urban settings.

## 2. Background and Rationale

Jakarta has been identified as the most populous city of Indonesia and also as the fourteenth most populous city of the Asian region. The diurnal variation in the metropolitan city ranges from 11 million during the daytime to 9.6 million during the night [34]. The current population density of more than 15,924 people per km^2^ is already quite dense [35], and the projected population of 11.4 million by 2035 can only be expected to constrain the limited biophysical resources of the city [36,37,38]. Along with such high population challenges, natural disasters commonly affect Jakarta.

Of the major natural disasters, Jakarta has a long history of devastating floods. In fact, flooding has remained a high concern since the city was named *Jayakarta* around the 17th century [39]. The oldest flood record is from 1621, when most of the city was under water, causing extensive economic loss for the Dutch colonial government at that time [40]. Floods erode development gains and affect large parts of the population in this capital city of Indonesia. In the coming years, more severe impacts of flooding are expected in Jakarta as a result of global climate change and human-induced factors [41]. With a combination of sea-level rise and land subsidence, the expansion of the potential flooded area deeper than 1.0 m is estimated to be around 110.5 km^2^ [42]. 

The consequences of the flooding of Jakarta have been serious. For example, floods in 2002, 2007, 2013, and 2014 led to tremendous direct and indirect economic losses. The 2007 flood killed 79 people, displaced more than 422,000 people, destroyed 1500 homes, and damaged many other facilities and infrastructure [43]. The estimated total economic loss for that single flood was around USD 695 million [44,45]. Furthermore, public health issues such as injuries and increases in sanitation-related diseases are important consequences of flooding in Jakarta [46,47].

In response to this, there has been much improvement regarding flood risk management in Jakarta. Since 2012, the Provincial Disaster Management Agency (*Badan Penanggulangan Daerah—*BPBD) has more effectively coordinated and managed disasters (including flooding) and most parts of the city’s river have been dredged [48]. Furthermore, the provincial government has focused its efforts on clearing the riverbank from settlements and moving people to safer areas. Despite such improvements, flooding remains problem an ongoing chronic problem in Jakarta and is considered as one of its main development challenges [49].

The need to build community resilience has been brought to the fore in Indonesia since the launch of the Hyogo Framework for Action (HFA) in 2005 [25]. Following its launch, the notion of “resilience” has been widely used in policy documents related to disaster management. In Jakarta, disaster resilience is stated as the central theme of the Provincial Disaster Management Planning 2013–2017 document [50]. Despite this, there is no common definition of resilience amongst stakeholders involved. Furthermore, study regarding priority needs in building community resilience to tackle flood risks remains limited.

The absence of common priorities of community resilience might create conflicts between stakeholders [51]. Though each stakeholder implements efforts intended to build community resilience, each relies on its own strengths and is focused on their own priority targets; some of these are complementary, whilst others are conflicting. Different stakeholders apply the resilience concept differently due to different priorities, areas of jurisdiction, and mandates [24].

The Jakarta Bay reclamation and giant floodwall mega-project provides examples of conflicting priorities and agendas that have triggered many long debates between different stakeholders [52]. The Jakarta authority believes that this project will benefit the safety of people and sustainable economic development in Jakarta, particularly in terms of preventing permanent floods [53] and improving the water quality in Jakarta Bay [54]. However, the National Disaster Management Agency has expressed concerns that this mega-project could worsen the flooding in Jakarta as it might slow the water flow from the drainage system to Jakarta bay areas [55]. Further, the Ministry of Marine and Fishery has argued that this activity would potentially disrupt the marine and coastal ecosystem and could result in massive economic losses in the fishery sector [56]. This fragmentation is a result of no institutional framework for collaboration between key stakeholders to build resilience [57]. A study has suggested that an organizational fragmentation is one of the ‘institutional traps’ that might challenge the process of resilience building [58].

The aim of this study is to investigate the priorities of community resilience against flood risks in Jakarta from the perspectives of three key stakeholder groups: DRR, CCA, and development. This study is important to elaborating the key elements for community resilience and providing an understanding of the common strategies that can be employed for building urban community resilience to flood risks across different groups. Specifically, it will identify the desirable characteristics of flood-resilient communities, as well as the key barriers to community resilience building from the three different stakeholder perspectives.

## 3. Methodology

### 3.1. Research Question

This study seeks to answer the following central question: What are the priority actions that can be taken to build community resilience against flood risks and to enhance synergy between DRR, CCA, and development groups in Jakarta? To answer this question, this study will address these two sub-questions:

1. What are the desirable characteristics of and influential barriers to community resilience against flood risks in Jakarta from the perspective of all stakeholder groups? and
2. What are the commonalities and differences between key stakeholders of each group in defining efforts to build community resilience to flood risks in Jakarta?

### 3.2. Study Method

This study applied a mixed method approach utilizing both quantitative and qualitative methods. This approach avoids exploration through one lens, and can thus reveal and help us understand a complex phenomenon [59]. Previous study has suggested that using both quantitative and qualitative methods is important for research triangulation [60].

The data collection process took place from 2014 to 2015 in Jakarta. There were two modes of data collection. They included quantitative data collection via a survey questionnaire, followed by qualitative data collection through key-informant interviews.

#### 3.2.1. Quantitative Data Collection and Analysis

The survey was administered in a paper and online format. A descriptive literature review was first conducted to aid in the design of the questionnaire. A descriptive perspective aimed at capturing respondents’ perceptions in two areas governed the development of questions used in the questionnaire across two aspects: (1) key features of a flood-resilient community and (2) key barriers to urban community resilience.

As can be seen in Appendix A, the pre-set questionnaires, based on the literature review output from Section 3.3.1 (regarding the twelve characteristics) and Section 3.3.2 (the nine barriers), were provided to the target groups. They were first chosen based on their active involvement in flood resilience building activities and then snowballing. For the online distribution, surveys were sent through existing networks, such as related professional email group lists and social media platforms. The respondents were asked to choose three characteristics that they believed were most needed and three barriers that were most influential to the situation in Jakarta.

The total number of survey respondents was 221, including 70 paper-based and 151 online surveys. The response rate for the paper survey was 35% (70 surveys were returned out of 200 that were distributed). For the online survey, it was not possible to calculate the response rate because of the distribution method described.

Respondents were asked to choose which group or stream best described themselves: the DRR, CCA, or Developmental group. The highest number of respondents chose the developmental group (97 respondents or 43.9%), followed by DRR (69 respondents or 31.2%) and CCA (55 respondents or 24.9%).

Descriptive statistics, including characteristics of community resilience against flood risks, and barriers to building a flood-resilient community, were used to analyze the questionnaire findings. The findings are presented for all combined respondents and each group (DRR, CCA, and Development). A simple statistical analysis (percentage) was used to describe the results. Frequencies of the data were counted to characterize the responses regarding features and barriers of community resilience to flood risks in Jakarta.

#### 3.2.2. Qualitative Data Collection and Analysis

It was difficult to identify the key informants for in-depth interviews. One reason for this is that, in Jakarta, DRR and CCA are not unique sectors in themselves and many stakeholders claim involvement in both fields. While it was impossible to interview all relevant stakeholders in Jakarta, using the researcher networks established prior to this research in Jakarta, the researcher approached the three main agencies: the Provincial Disaster Management Agency (BPBD), the Provincial Environmental Management Agency (BPLHD), and the Provincial Development Planning Agency (BAPPEDA), whose main duties relate to DRR, CCA, and development, respectively. This allowed researchers to identify and determine the key informants suited to the needs of the research through existing networks [61].

Twenty-six key informants representing their institutions were included (see Table 1). Key stakeholders were defined as a member of an organization who was actively involved in a DRR-, CCA-, or development-related program in Jakarta, including the provincial government, international institutions, the Red Cross and Red Crescent movement, community-based organizations, private institutions, multi-platform institutions, research institutions, and non-governmental organizations.

Quotes from the interview process were used to provide more detailed information and descriptions for the findings from the questionnaire. Quotes are provided verbatim, although the authors have used square brackets [xxx] or dots (…), which respectively indicate edits and text that has been removed for the conciseness of the displayed data.

As for the qualitative data analysis, as suggested by previous research, thematic analysis was conducted to extract detailed information on the complex phenomenon of achieving community resilience to flood risks [62].

### 3.3. Designing the Questionnaire

**Figure 1:**
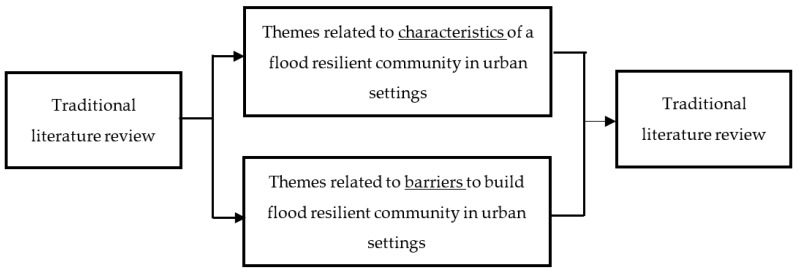
Flow of the questionnaire design process.

A traditional literature review was conducted in order to design the questionnaire to elicit the characteristics of and the barriers to an urban flood-resilient community. This was necessary for understanding what had already been identified as common characteristics of and barriers to community resilience in the context of urban settings in developing countries (see Figure 1). The primary criterion of inclusion-exclusion to search the articles was relevance. To ensure that the articles were relevant, the study only included articles that were relevant to community resilience against flood risks (or climate-related hazards) in urban (or peri urban) settings.

Combinations of keywords were used, such as ‘flood’, ‘resilience’, ‘urban’, ‘community’, ‘characteristics’, ‘barriers’, and ‘challenges’. As well as sourcing peer-reviewed literature from databases such as Scopus and Google Scholar, the general Google search engine was also used to capture relevant grey literature documents (e.g., policy documents, guidelines, and reports released by credible relevant organizations).

#### 3.3.1. Themes Regarding Characteristics of an Urban Flood-Resilient Community

While there is no single definition of ‘resilience’, the term itself comes from the Latin word, *resilio*, which means jumping back [63]. Within the context of a disaster and community, previous authors have developed a working definition of resilience, which is “*the capacity or ability to anticipate, prepare for, respond to, and recover quickly from impacts of disaster”* [64], p. 4. Another authors further explained that resilient communities “*are bound to feel empowered, less vulnerable, more content with their environment and prepared to face situation of crisis*” [65], p. 52.

It is becoming increasingly recognized that resilience is conceptualized within specific contexts and uniquely tailored to the targeted setting [66]. As a result, a number of authors have suggested that a resilience concept needs to be framed to the local context [67,68,69]. From existing literature, this study identified 12 themes for characteristics of a flood-resilient community in urban settings of developing countries (see Table 2).

#### 3.3.2. Themes Regarding Barriers to Urban Community Resilience against Flood Risks

Understanding barriers is critical to enhancing our understanding of developing resilience. ‘Barrier’ here is defined as anything that might hinder the process of urban community resilience building. The barriers to community resilience building are expected to differ from one setting to another. In the context of flood risks in urban settings, this study identified 11 common themes from existing literature, as shown in Table 3.

## 4. Results and Discussions

### 4.1. Urban Flood-Resilient Community in Jakarta: Characteristics and Barriers

The first question in this study sought to determine the concept of community flood resilience in the context of Jakarta through understanding its desirable characteristics and influential barriers. As discussed earlier, each setting is unique and there is no ‘one size fits all’ approach for community resilience building, so community resilience building has to be tailored accordingly [100]. The authors have discussed the top three desirables and top three barriers in achieving community resilience to flood risks in Jakarta, from the above-mentioned options presented to the survey respondents.

#### 4.1.1. Desirable Characteristics for Community Resilience towards Floods Risks

**Figure 2:**
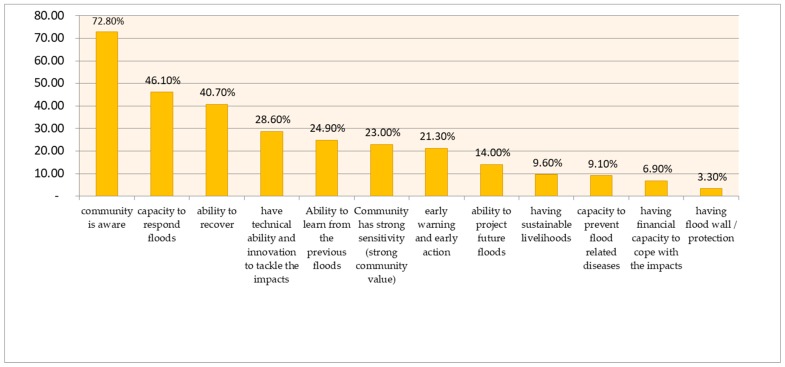
Combined responses of respondents’ views on characteristics of a flood-resilient community in Jakarta.

Based on the combined responses of all groups, as shown by Figure 2, most respondents (72.8%) identified the community that has flood risk awareness and knowledge as the most desirable. This was then followed by a community that has the capacity to respond (46.1%) and a community that has the ability to recover (40.7%).

##### Community that is Aware of Their Flood Risks

This finding indicates that being flood resilient is highly influenced by the level of awareness of the people. This is consistent with the findings previous authors who recommended that flood risk awareness is key for the governance of flood risk reduction, particularly in the era of a changing climate in Jakarta [101].

Several reports have shown that community awareness of flood risk means understanding the hazard, the exposure, the vulnerability factors that might exacerbate the impact, and the capacity that can be utilized to prevent or reduce loss and damage [50,102]. Flood risk awareness will bring full consciousness to each citizen and will have a positive implication for the improvement of risk communication to avoid or minimize the impacts [82]. A key informant seconded the important role of community awareness:

“Flood risk awareness and knowledge is important as this will enhance sense of alertness of the community members … sense of alertness towards flood risks will become the foundation of community preparedness” (INS-11).

In Jakarta, many factors might increase the flood vulnerability, such as people living in flood-prone areas, limited access to safe drinking water and sanitation, improper urban infrastructure planning, and a lack of waste management [103,104]. Furthermore, an excessive use of ground water that triggers land subsidence is also another ongoing problem in Jakarta [36,105]. Previous authors have confirmed that lack of community awareness of flood risks has made Jakarta more vulnerable to flooding. Additionally, they suggested that community awareness should be the priority to enhance the resilience level of Jakarta to urban flooding [103]. This was also supported by one key informant:

“Community that is aware of flood risks means they understand the factors that might worsen the flood impacts. Which most [of these factors] are strongly related to human factors themselves” (INS-25).

Previous study has obtained a closer view of the implementation of Ciliwung Watershed Ecosystems Management and Restoration (Ciliwung WEMR), a National Government-led program that aims to mitigate flooding through a comprehensive river ecosystem approach [106]. They found that collective awareness of the people who live near the watershed is one of the pillars of this program determining its success.

Moreover, previous authors have demonstrated that a community with a high flood risk awareness will be actively involved in local adaptation measures to limit the impact of flooding in Jakarta (such as participating in communal work to clean drainage) [107]. Consistent with this, another author who conducted research in 43 coastal communities in Indonesia and discovered that community members that are aware of their local disaster risks are the ‘backbones’ of response activities whenever disasters strike their localities [108].

##### Community that has the Capacity to Respond to Floods

As flood risks, to some extent, are inevitable, the capacity to respond to risks is important for preventing life loss and injuries and preserving properties [102]. The National Strategic Planning for Disaster Management Agency has stated that the capacity of community members and local governments to respond to disaster events is key to increasing disaster resilience [109].

How early and appropriate the response of the community to the incoming floods is, are influenced by information dissemination from the authorities. Previous authors have noted efforts by the BPBD to enhance the response capacity of the community, such as the establishment of a digital flood information management platform (namely PetaJakarta.org) [110]. Another author confirmed that this online platform is useful not only for disseminating real-time situational data regarding flood conditions, but also for harnessing reliable and relevant information from the social media (Twitter) accounts of Jakartans [111].

A prior researcher conducted an anthropological analysis at several flood-prone *Kampung* in Jakarta. He found that community members have some common measures for coping with flood risks, such as preparing ready-to-eat meals and drinking water for emergencies, having a ladder at their house to climb to the roof, having knowledge about the evacuation procedure, and keeping valuable documents in a place safe from floodwater [48].

Community participation is the ‘soul’ of an effective response to a flood event [112] and is maintained through a documented community contingency plan, as identified by a key informant: 

“This disaster contingency planning belongs to the community in each particular suburb. The (Disaster Management) Agency only facilitates the community to develop contingency plan. We provided them with the guidance, but most of the ideas inside the plan originated from the community members” (INS-02). 

A community contingency plan should include data and information about the vulnerability factors, responsibilities of each key local actor, need assessment analysis, inventories of relevant capacities to respond, and consensus on a list of priority activities during the emergency response. A contingency plan is a modality for risk communication and preparing disaster recovery processes at the community level [113].

##### Community that has the Capacity to Recover

The recovery capacity is the ability to reconstruct the system after the flood event [102]. Flood impacts in Jakarta are mostly related to economic or financial implications. In relation to this, previous authors have commented that one of the hardest challenges to dealing with the recovery process in the aftermath of flood events is to address the direct and indirect economic damages [114]. It has been suggested that floods do not equally impact all households and the low socio-economic families will be impacted the most [115]. This is in line with one informant:

“The rich don’t bother too much with the flood as they have all means to support the recovery process, however those who have not, some will have to experience a long recovery process, and some will even fail to return back to normal” (INS-09).

However, as identified by previous authors, even when financial access is limited, strong social capital (such as the application of a traditional Indonesian communal work system, namely *gotong* royong) can fuel post-flood recovery activities in neighborhoods throughout Jakarta Province [107]. Similarly, in the aftermath of the 2011 Brisbane flood, strong social capital was one of the main factors that sped up the post-flood recovery process, particularly in cleaning up the affected areas [116].

#### 4.1.2. Influential Barriers to Community Resilience Building Efforts

**Figure 3:**
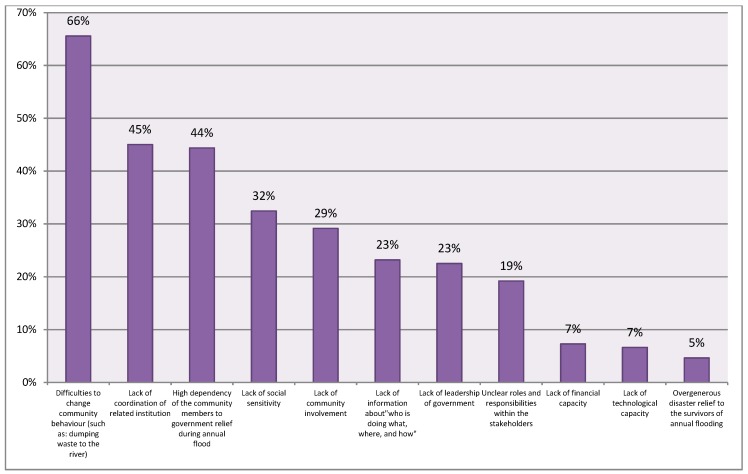
Combined responses on perceived barriers to urban community resilience building in Jakarta.

Figure 3 shows the combined responses from all groups. From this figure, most respondents believed that “difficulties in changing community behavior” was the most influential barrier (66%). The second and third rated barriers were “lack of coordination of related institution” and “high dependency of the community members to government relief aid”, with values of 45% and 44% respectively. The following sections will elaborate these findings.

##### Difficulties in Changing Behavior

Previous research has established that rapid urbanization coinciding with human economic activities and a lack of environmental awareness can lead to increased vulnerability to hazards, particularly urban flooding in Jakarta [117]. It has been observed that dumping waste into the river would decrease the capacity of drainage systems due to blockage, which would then lead to the overflow of riverbanks. Further, dumping waste would worsen the health impact of floods due to the polluted floodwater. The National Development Planning Agency (Bappenas) confirmed that about 96% of the river water in Jakarta is heavily polluted [118].

In relation to this poor behaviour towards the environment, a study has confirmed that inappropriate waste dumping by riverbank settlers not only increases flood risks, but also creates many other environmental problems [119]. Furthermore, another report also explained that the carrying capacity of the Ciliwung River (one of the major rivers in Jakarta) has declined due to economic activities conducted by the people around the river [117]. Moreover, it has been observed that this community behavior challenge, combined with the lack of an effective waste management system and weak law enforcement, increased the flooding impact and is consistent with findings that have demonstrated that human activities are a major factor that can worsen flood impacts in Jakarta [103].

In addition to this, prior study has found that the waste collection fee imposed is a burden for low-income families and they prefer to dump their domestic waste in the river [120]. In regards to this financial incapability, some families were reluctant to use the water from the council to avoid payment and chose to live with a public health risk owing to the usage of contaminated river water for daily basic needs [118].

A key informant who works for a community owned organization that is concerned with the protection of the Ciliwung River identified that, to some extent, the community has contributed to the maintenance of the river using their own resources, but that they required extra support from the relevant authorities to make their efforts more sustainable:

“To solve this problem, we cannot always point finger to the community members. Yes, some of us are still doing this bad habit (dumping waste to the river), but some are aware of the importance of keeping our river clean thus we organize a collective action to clean up the river and encourage the others to do the same. Here, we need the authorities to support our initiatives” (INS-14).

However, according to one of the key informants, the implementation of enhancing disaster risk awareness at a community level is challenged by limited budget allocation for disaster preparedness, particularly promotional activities such as disaster risk awareness campaigns:

“We understand the importance of raising community awareness, however we could not do much as the budget is limited. There is more budget allocation for disaster response, but less for prevention and preparedness unfortunately” (INS-02).

##### Lack of Coordination across Related Institutions

Respondents voted a lack of coordination across related institutions as the second most influential barrier for urban flood resilient community building. The coordination of efforts between actors when responding to disaster events is crucial to avoiding unnecessary duplication and dealing with a lack of resources and capacity constraints [121]. Previous researcher has advised that policy coordination across agencies is the factor that can enable a region to adapt to climate change impacts [122].

In regards to this finding, prior research has suggested that a lack of coordination amongst key actors of flood governance in Jakarta has led to a shortness of data and information sharing [38]. In addition, a number of researchers conducted a study to evaluate Jakarta’s flood defense governance and discovered that the ineffective coordination between provincial and district authorities, combined with unclear roles and responsibilities between key governmental institutions, has caused delays in the implementation of flood risk management activities [123].

Previous studies have explained why a lack of coordination of related institutions has become a persistent issue for the governance of flood risk management in Jakarta. It has been found that a lack of interaction and communication between related key actors seems to be one of the main causes of this coordination issue [107]. As described by prior studies, another possible reason is the policy and institutional fragmentation within the arena of flood risk management in Jakarta [124,125].

Related to this, a key informant mentioned the following:

“Every agency views resilience from their own perspectives. The bottleneck is that all agencies are fragmented … coordination line for sharing ideas and information does not work” (INS-07).

Furthermore, one informant identified that the coordination line is ineffective for triggering partnership and collaboration between actors due to the existence of ego-centrism: 

“Everyone want to be recognized as the key player in flood risk reduction in Jakarta. So, it is like a competition. Each only focused on how to complete their own tasks thus coordination with others is not that important for them” (INS-18).

##### High Dependency of Community Members on Government/External Assistance

The next most influential barrier for community resilience building in Jakarta is the high dependency of community members on external aid. It is important to note that the concept of community resilience is not only about the ability to bounce back, but also bounce forward, which requires the community to be self-reliant to cope with disaster risks [126].

The government funding could either support the community to build their resiliency or impede the process [90]. To ensure the former, the government should provide opportunities to the local community members to activate their own contingency planning when flooding strikes [77]. The community members should also be pro-active in using their flood contingency planning to limit the impacts of floods. A community participation approach is necessary for the Jakarta Government to design a strategy to deal with floods [47].

The Jakarta Provincial Government is aware of the importance of community participation and has clearly stated in their Disaster Management Plan that a recovery process in the aftermath of a disaster event should fully involve affected community members [50]. As an example, provide an example of this: during the 2007 flood, the community members in Bukit Duri (one of the most flood-prone suburbs in Jakarta), together with local NGO, optimized their own resources to help with the evacuation process and set up emergency kitchens, and were thus able to feed the affected community members for 4 days [127].

However, one informant made the following comment:

“Some people are just too generous. Sometimes the flood only impact a small amount of households, still many external organizations come directly to the affected community and provide them with foods, blanket, rice, etc..,. Even sometimes the National government also provide relief items directly to the community members although the Jakarta government is actually can handle them. This create chaotic situation and spoil the community members” (INS-02).

Another key informant added the following:

“Because the affected communities know that people will come to help them, they become passive and just wait for somebody to deliver food to their doorsteps” (INS-19).

From these interviews, we can argue that dependency mentality is not merely a cultural factor, as described by previous authors this could be also a result of uncoordinated disaster relief operations [128]. Unplanned excessive relief aid can harm the community and create dependency among the community members that will hinder the overall recovery process [129].

### 4.2. Understanding Commonalities and Differences between Groups

Contrary to the expectations presented in previous studies, e.g., [57,130], the survey findings did not reveal a significant difference in views between DRR, CCA, and Development groups in regards to desirable characteristics and influential barriers of flood-resilient communities in Jakarta.

**Figure 4:**
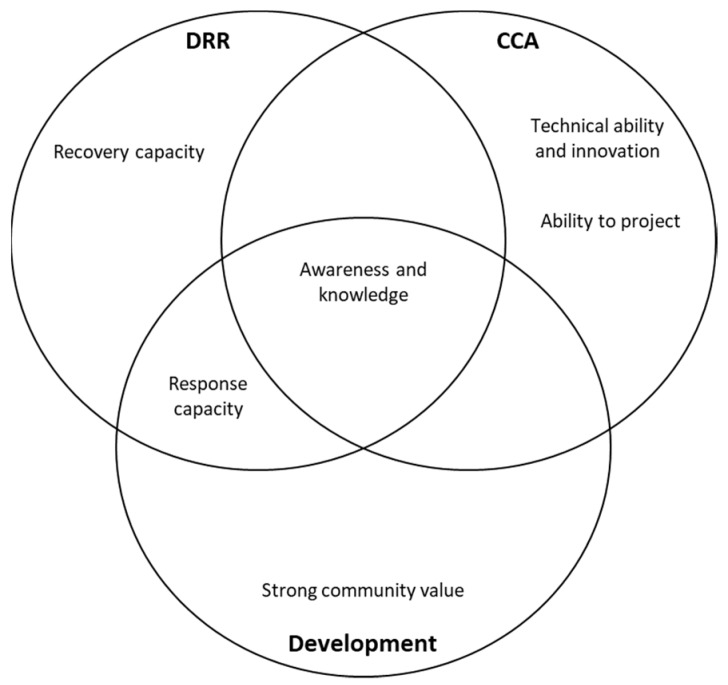
Similarities and differences in stakeholders’ views of the three most desirable characteristics of community resilience.

Regarding the desirable characteristics for flood resilience, flood risk awareness and knowledge were the highest for all groups (Figure 4). This finding shows that DRR, CCA, and Development groups acknowledge the importance of risk awareness and knowledge in the process of community resilience building in Jakarta. As for the differences among the three groups, it was only the CCA group who viewed technical ability and innovation and ability to be amongst the top three most important features of community resilience against flooding in Jakarta. On the other hand, the DRR and Development groups identified recovery capacity and strong community values, respectively, as being in the three most important features of urban community resilience against flooding in Jakarta.

The CCA group observed that technical innovation and adjustment strategies have been implemented by people in Jakarta to adapt to flood risks, such as by raising the house level, building terraced housing, and building dikes to prevent flood water from coming inside their houses [107]. The DRR group provided views consistent with the Jakarta Disaster Management Planning document, which stated that the community is the backbone of the response and recovery process in the aftermath of disaster events [50]. Community capacity, in the Indonesian context, is closely related to the spirit of *gotong royong* (mutual assistance) which is required to build a society in Indonesia. Furthermore, prior research has demonstrated that a strong community value speeds up the flood recovery process [131]. 

**Figure 5:**
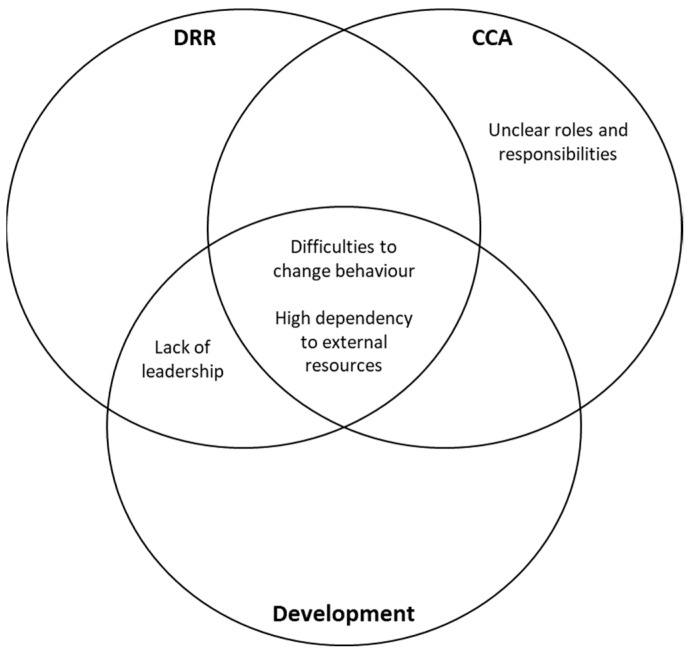
Similarities and differences in stakeholders’ views of the three most influential barriers for community resilience.

Regarding barriers, the three groups agreed that improving community behavior and removing a dependency mentality from the community are amongst the top three influential barriers in building community resilience towards flood risks in Jakarta (see Figure 5). It was only the CCA group who identified unclear roles and responsibilities within the stakeholders as being in the top 3. This is in-line with the beliefs expressed by a CCA informant (here represented by the Environmental Management Agency of Jakarta Province) around a lack of mandate to govern and lead the adaptation field, thus the division of roles and responsibilities between stakeholders is not clear.

Despite the fact that there are more commonalities than differences among DRR, CCA, and development groups, one key informant believed that flood resilience building is still seen from a sectoral and fragmented perspective in Jakarta. Moreover, another interviewee also noted the disconnection between the policy umbrella of each group, which discouraged them to collaborate. Without clear guidance or policy to support cross-sectoral actions, working in silo is likely to continue. As identified by one key informant,

“The bottleneck is that all agencies are fragmented. Coordination line does not work. Integration is easier said than done*. Ego sectoral is also a persistent issue here, every stakeholder has a desire to raise their own flag*” (INS-07).

## 5. Discussions and Recommendations: The Way Forward

This study identified two important priority actions for building community resilience in Jakarta, which are (1) building urban community resilience from the people (or people-centered urban resilience) and (2) strengthening collaborative governance to facilitate partnership between relevant actors. The following section will discuss these two points.

### 5.1. Paradigm Shift: Building Community Resilience from ‘The People’

Despite the agreement of most stakeholders that human and social factors are the fundamental risk drivers of floods in Jakarta, it has been reported that, unfortunately, the solutions to address flood risks were still dominated by an engineering-based approach and did not comprehensively consider the needs of the targeted communities [53]. This rather contradictory result may be due to the interviews focusing on the ideal application of community resilience as a policy narrative in Jakarta and not about the actual implementation. Therefore, although stakeholders believe in the importance of transformational adaptation, the incremental or conventional type of adaptation remains the main option in Jakarta. This result may be explained by the fact that conventional flood adaptation through structural mitigation is preferable as it provides tangible or quantifiable outcomes which can be easily measured (e.g., river dredging, dyke construction, coastal protection). A key informant also mentioned that flood mitigation financing schemes always need verifiable indicators to measure the effectiveness of the program. Adopting a physical infrastructure approach is a popular option for flood risk reduction projects. Another possible explanation as to why infrastructure solutions are more popular in flood resilience building is that resilience thinking has been in the discourse of built environment professionals for longer, compared to the social and humanities fields [132].

**Figure 6:**
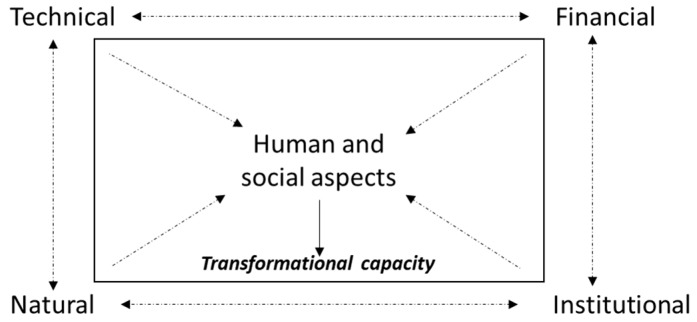
People-centered resilience building: Multi-dimensional approach with human and social in the center.

To achieve transformational urban flood resilience, this study suggests that it is necessary for the Jakarta Government to shift their paradigm from a technocratic approach towards a people-centered one which includes human and social aspects (see Figure 6). As indicated by previous study, to make a city more resilient to climate-related disasters, a win-win solution is required that is comprehensively understood within the roots of urban complexities and applies multiple interventions to address those problems [133]. By focusing on the people, it does not mean that the roles of structural mitigation in reducing flood impacts in Jakarta are disrespected; however, it is equally, if not more, important for the Jakarta Government to invest more in non-structural mitigation, especially through human and social aspects, particularly knowing that community behavior is the central issue that contributes to the extent of flood damage in Jakarta [103].

Therefore, although it is still necessary to consider all aspects in building community resilience like suggested by previous researcher [64] our findings reveal that, in the context of a complex megapolitan city like Jakarta, human and social aspects lay the basic foundation of transformational capacity, which is a critical milestone for advancing the degree of transformability of the community. Transformability here is defined as the “*collective capacities to create a fundamentally new system when ecological, economic or social conditions make the existing system untenable*” [134], p. 744. It can thus be suggested that no flood risk reduction project should leave out the human and social dimensions, such as community awareness raising and social infrastructure building. This finding is similar to what was presented by previous literature who identified the function of human and social infrastructure as the main ingredient of community resilience in the aftermath of Superstorm Sandy [135]. The literature confirmed the relationship between collective efficacy and social exchange, which cultivated the capacity of the community members to prepare for and recover from disaster events [135]. Another study from a different setting also demonstrated that social cohesion is essential to speeding up the recovery process within the affected communities after two major earthquakes (magnitude 7.8 and 7.6) hit Nepal in 2015 [136].

To implement this people-centered approach, previous literature has noted that, Jakarta Government should focus on activities that promote collective responsibilities and stakeholder participation, as well as improve risk communication and institutional capacity building [89]. Behavioral improvement will require full awareness of the community members for it to be sustainable. Community members that have a full awareness of their disaster risks are more likely to participate in disaster preparedness activities and less likely to collapse when recovering from a disaster [137]. This finding has important implications for the authorities in Jakarta when attempting to create a risk-informed society as an output to achieve a flood-resilient community. In relation to this, the notion of ‘risk-informed’ was highlighted in the statement of the Special Representative of the Secretary General for DRR, Miami Mizoturi, to commemorate the International Day for DRR in 2018. She noted that, “if it’s not risk-informed, it’s not sustainable, if it’s not sustainable it has a human cost” [138], p. 1.

### 5.2. Collaborative Governance for Effective Synergies between Streams

The quantitative findings elucidated the views of a large number of relevant stakeholders on the conceptual ideas of a flood-resilient community in Jakarta. It found many commonalities across the groups regarding the characteristics of and potential barriers to community resilience. This knowledge could become a shared motivation for integration between DRR, CCA, and Development agencies. Contrary to this expectation, the qualitative findings showed that collaboration between key groups is still not an easy task. This result may be explained by the fact that there is no policy umbrella for a flood-resilient community, and this is necessary not only to provide policy direction, but also to ensure the compliance of all key stakeholders to collaborate with different agencies. Another possible explanation for this is that many, if not most, flood resilience projects (particularly those under DRR and CCA groups) are donor-driven, which means that they are often too rigid and not flexible enough to be amended or interconnected with other projects from difference sources of funding. This is similar to prior researchers in the Philippines who discovered that the decision-making process of any donor-driven project is complex due to the dynamic interactions between donor agencies (which are mainly from foreign countries), the national government, and the provincial/local government [139]. Furthermore, they also confirmed that involvement of power play factors complicates the matter.

In 2017, the National Disaster Management Agency (BNPB) and Ministry of Environment and Forestry (KLHK), with support from the United Nation Development Program (UNDP), launched the DRR and CCA convergence framework. This relatively new framework proposes DRR and CCA convergence in five different dimensions: policy, institution, budget, project management, and methodology [140]. Even though this convergence framework exists, it has been reported that multi-sector coordination at the local level remains an issue. Weak leadership is the factor behind ineffective coordination between different stakeholders at local levels. This reflects that a policy framework for integration will not be functional without effective leadership to facilitate the collaboration [122].

In response to this issue, this study proposes the use of collaborative governance as an appropriate way forward, which, according to the literature, is effective for addressing ‘wicked issues’ that require a multi-actor, multi-level, and multi-sector solution approach, e.g., [141,142]. The collaborative governance framework consists of three fundamental dimensions: collaboration dynamics (principled engagement, shared motivation and capacity for joint action), a collaborative governance regime, and system context [143], p. 12. System context, which in this regard is community resilience building, is defined as the surrounding environments that will affect and be affected by the collaborative governance regime. For this reason, community resilience building must be seen from a holistic perspective and failure to do so will lead to disconnected policies and disharmony between different institutions. As this paper brings to light the perspective of each group regarding features and barriers to a flood-resilient community, it is expected that the existence of a DRR–CCA convergence framework could be more meaningful and useful to establish the three fundamental dimensions (as listed previously) for collaborative governance to implement a community resilience program in Jakarta Province.

Finally, the limitations of our study must be acknowledged. The sample size for the quantitative survey data analysis was less than the goal identified through sample size calculations. Due to this, the authors could not conduct a statistical comparative analysis of perspectives exhibited by different groups (DRR, CCA, and developmental groups). However, these limitations should not have significantly impacted the quality or outcomes of this study, as the authors were confident in the high-quality data from all key stakeholders involved in the process of DRR and CCA policy development in Jakarta. Another limitation to note is that this research did not elaborate the utilities of collaborative governance concepts, so further work should be undertaken to investigate the opportunities and challenges of collaborative governance regimes in integrating DRR, CCA, and Development groups.

## 6. Conclusions

This study set out to understand the main features of community flood resilience in urban settings and the common priorities of DRR, CCA, and Development groups in regards to flood resilience building in Jakarta. The findings have important implications for guiding the development of urban flood resilience strategies, particularly in terms of resource allocation and priority settings. This study can help decision-makers to identify the potential partnership glue factors by better understanding the commonalities and differences between the three groups.

While the literature identifies the importance of human, technical, financial, natural, social, and institutional aspects in efforts to strengthen community flood resilience, the findings from this study reveal that the majority of stakeholders surveyed believed that urban flood resilience building in Jakarta has to be centered upon the human aspect (including the element of a risk-informed society). This paper urges that, for any city with complex environmental and societal problems, like Jakarta, this shift of thinking should be adopted in their flood risk reduction policies, programs, and activities.

Although the three groups shared the same views about urban flood resilience, they were still working under completely different program planning frameworks without formal and structured coordination and communication mechanisms. It is imperative that future policy and planning actions permit all stakeholders to work in partnership to build resilience. To cultivate the spirit of partnership, a collaborative governance regime is proposed by this paper. This should be coupled with effective leadership at the provincial government level to facilitate such collaborative work. 

It is hoped that increasing the understanding of each group’s perspective in dealing with flood risks will not only enhance the effectiveness of the flood resilience building process, but also provide simultaneous benefits for social systems coping with challenges posed by climate extremes and climate change.

## Figures and Tables

**Table 1:** Background, coding, and grouping used for key informant interviews.

Background	Code	Grouping	Background	Code	Grouping
Provincial Government	Ins-01	Development	Community-based organization	Ins-14	CCA
	Ins-02	DRR		Ins-15	DRR
	Ins-03	Development	Private institutions	Ins-16	DRR
	Ins-04	Development		Ins-17	Development
	Ins-05	Development	Research institution/University	Ins-18	DRR
	Ins-06	CCA		Ins-19	CCA
	Ins-07	CCA		Ins-20	CCA
International institutions	Ins-08	Development		Ins-21	DRR
	Ins-09	Development	Non-governmental organization	Ins-18	DRR
	Ins-10	DRR		Ins-19	CCA
Red Cross and Red Crescent movement	Ins-11	Development		Ins-24	DRR
	Ins-12	Development		Ins-25	CCA
	Ins-13	Development		Ins-26	DRR

**Table 2:** Themes for characteristics of a flood-resilient community in urban settings of developing countries.

Characteristics	Key Themes	Author(s)
Flood-resilient community in urban settings in developing countries is a community that …	Has flood risk awareness and knowledge	[70]
	Has the ability to project future floods	[71]
	Has the ability to respond	[41,72,73]
	Has technical/innovation solutions to mitigate the impacts	[74,72]
	Has the ability to recover	[41,75]
	Has the ability to learn	[76]
	Has access to resources	[77,78]
	Has knowledge regarding public health preparedness	[79]
	Has sustainable livelihoods	[80,81]
	Has engagement in early warning systems	[82,83]
	Has a strong social connectedness	[84,85,86]
	Has physical protection measurement	[74,87]

**Table 3:** Barriers to urban community resilience to flood risks.

Barriers	Key Themes	Author(s)
Factors that could hinder the implementation of community resilience against flood risks in developing countries are …	Lack of financial capacity	[87,88]
	Lack of technology	[89]
	Poor habit of communities (e.g., dumping waste in rivers)	[90,91]
	Lack of coordination across related institutions	[92,58]
	Lack of leadership	[58,93]
	Unclear roles and responsibilities within the stakeholders	[58]
	Lack of information about who is doing what, where, and how	[94]
	Community has little social sensitivity	[95]
	Lack of community involvement/participation	[96]
	Overgenerous disaster relief to the survivors of annual flooding	[97]
	High dependency of the community members on government relief during annual floods	[98,90,99]

## Appendix A. Questionnaire used for data collection

The following questions are about defining urban community resilience to floods in Jakarta:

1. From your point of view, which features of urban community resilience that are desirable in the context of floods in Jakarta (Please only select a max of 3 from the 12 options below):
  Community is aware of flood risks and hazards	☐1
  Has capacity to respond floods	☐2
  Has ability to project future floods	☐3
  Has capacity to prevent flood related diseases	☐4
  Ability to recover after floods	☐5
  Ability to learn from the previous floods	☐6
  Has sustainable livelihood	☐7
  Having technical ability and innovation to tackle the impacts	☐8
  Having financial capacity to cope with the impacts	☐9
  Involve in early warning and early action	☐10
  Has strong sensitivity (community value)	☐11
  Has flood wall protection	☐12
2. Choose three of statements below describe best the problem that Jakarta is currently facing: (Please only select a max of 3 from the 11 options below)
  Lack of funding	☐1
  Lack of technology	☐2
  Poor habit of the community	☐3
  Lack of coordination	☐4
  Lack of leadership	☐5
  Unclear roles/responsibilities of relevant stakeholders	☐6
  Lack of information sharing (who what where and how)	☐7
  Lack of social sensitivity	☐8
  Lack of community participation	☐9
  Overgenerous disaster relief/aid	☐10
  High dependency of the community members to government support	☐11
3. Do you have more comments regarding urban community resilience to flood risks in Jakarta?
4. From which institution/department are you?
5. Which stream describe your institution the best?
  Disaster risk reduction (DRR) stream	☐1
  Climate change adaptation (CCA) stream	☐2
  Both DRR and CCA/development	☐3
